# Personalized versus non-personalized computerized decision support system to increase therapeutic quality control of oral anticoagulant therapy: an alternating time series analysis

**DOI:** 10.1186/1472-6963-4-27

**Published:** 2004-09-29

**Authors:** Isabelle Colombet, Alessandra Bura-Rivière, Rémy Chatila, Gilles Chatellier, Pierre Durieux, for the PHRC-OAT study group

**Affiliations:** 1 Public Health and Medical Informatics, Georges Pompidou European Hospital, Paris, France; 2 Clinical Investigations Center, Georges Pompidou European Hospital, Paris, France

## Abstract

**Background:**

The quality control of oral anticoagulant therapy (OAT) during the initiation and maintenance treatment is generally poor. Physicians' ordering of OAT (especially *fluindione *and *warfarin*) can be improved by dose adjustment algorithms, taking into account the results of International Normalized Ratio (INR). Reminders at the point of care, computerized or not, have been demonstrated to be effective in changing physicians prescription behavior.

However, few studies have addressed the benefit of personalized reminders versus non personalized reminders, whereas the personalized reminders require more development to access patient record data and integrate with the computerized physician order entry system.

The Hospital Information System of George Pompidou European Hospital integrates an electronic medical record, lab test and drugs order entry system. This system allows to evaluate such reminders and to consider their implementation for routine use as well as the continuous evaluation of their impact on medical practice quality indicators.

The objective of this study is to evaluate the impact of two types of reminders on overtreatment by oral anticoagulant: a simple reminder of text formatted dose adjustment table and a personalized recommendation for oral anticoagulant dose and next date of INR control, adapted to patient data. Both types of reminders appear to the physician at the moment of drug ordering.

**Methods:**

The study is an alternating time series experiment with three 6 months periods, each one including every 2 months according to a Latin square scheme: a control period without any reminder, a period with the simple non personalized reminder, a period with personalized reminder. All patients hospitalized in departments using the computerized physician order entry system and ordered *fluindione *or *warfarin*, will be included in the study between November 2004 and May 2006.

Main outcome will be the proportion of overcoagulation, as expressed by the proportion of observation time with INR over 4.5, assuming INR change linearly. Secondary outcome is the incidence of major haemorrhagic events. Data will be collected thanks to Hospital Information Systems databases.

Data will be analyzed taking into account patient and physician clustering effect.

## Background

### Iatrogenic effects of oral anticoagulants therapy

According to a study carried out by French pharmacovigilance centres, haemorrhage subsequent to oral anticoagulant treatment (OAT) is the most common drug-related side effect resulting in hospitalisation in public hospitals in France (13% of such admissions, and 0.41% of the 3,137 admissions analysed). On the basis of these findings, the AFSSAPS (French Agency for Health Product Safety) has made the prevention of iatrogenic effects related to OAT one of its priorities. Many of these events are consequences of interactions between different drugs, resulting in inappropriate doses [1-3].

Implementation of a system of support when prescriptions are made out is likely to improve prescription practices and to decrease the frequency of side effects. It should be possible to integrate a support tool into the drug prescription system, by using nomograms to adjust OAT doses.

### Decision-making tools and appropriate practice

The efficiency of reminders issued at the time of prescription has been demonstrated by a various studies [4-8]. These reminders can come in a paper, telephone [9] or computer form. Hunt *et al*. [10] reviewed all studies (randomised and quasi-randomised) evaluating the effect of computer-based clinical decision support systems on medical practice and treatment outcome. They showed that these systems are effective for drug prescription purposes and for the implementation of various medical strategies [10]. We have demonstrated the efficiency of reminders in the French situation, both in the form of "paper" reminders [11] and in the form of computer-generated reminders [12]. Computer-generated reminders appear the most promising given the development of computer programs including prescription aids for hospital usage.

Several types of reminders can be issued at the time of prescription [13]:

• simple, general information concerning the recommendations that should be taken into account (non-specific or non-personalised reminders),

• "check list": includes questions or a precise list of practices that the doctor must tick to show that it has been done,

• reminders including clinical data concerning a specific patient that must be taken into account for a given procedure (personalised reminders).

The advantage of personalised reminders over non-personalised reminders has not been demonstrated in the literature. However, the production of personalised reminders necessitates better integration of existing information and is thus more expensive to develop. It is important to determine whether this personalised tool results in a better quality of care than non-personalised tools.

Several randomised clinical trials have tried to evaluate decision support systems for the prescription of OAT, but failed to draw any conclusions about their efficiency for several reasons: heterogeneity and complexity of the systems evaluated, experimental designs difficult to apply and not necessarily adapted, and too few patients included [14-17].

Reminders issued at the time of prescription have been shown to be effective by experimental studies, but the difficulties of maintaining the effectiveness of interventions designed to improve clinical practices remains a major problem. We evaluated the effect of an active decision support system for the prescription of low molecular weight heparin as prophylaxis for venal thrombosis in an orthopaedic surgery department. In this study, the system was and was not used during alternate periods. It showed that such programs affect practices without affecting learning [12]. Other authors have looked at the problem of the routine use of computerised systems [18,19]. The long-term use of a computer program to modify medical practices necessitates a hospital information system that ties in prescriptions and the results of medical tests, and the continuous collection of indicators making it possible to evaluate use and effect.

### Georges Pompidou European Hospital (GPEH)

#### Organisation of the information system at GPEH

The hospital information system currently collates prescriptions and results of biological tests and imaging procedures. Eight hundred computers, both laptops and fixed posts, are used to in care procedures (in care departments and medical offices).

The Dx-Care^® ^program is at the centre of care delivery. It is used by doctors and nurses:

• to prescribe laboratory examinations and imaging tests for a patient,

• to visualise the results of laboratory tests,

• to establish and to consult nursing schedules,

• to archive a structured observation,

• to prescribe drugs.

Dx-Care^® ^is integrated with other applications to allow circulation of information between departments, laboratories and the pharmacy. Prescriptions for laboratory tests are transmitted to the Netlab^® ^program which manages such tests (this program is used by all biochemistry, haemostasis and immunology laboratories, etc.) The laboratories return the results using this same program, which retransmits them to Dx-Care^®^. Furthermore, prescriptions of drugs are transmitted to the Phedra program, which is used by the pharmacy to manage prescriptions. The prescription is validated by the pharmacy and this validation is then transferred to Dx-Care^®^. The lab test prescription facility has been available in the hospital information system since 2000 and is used by all departments of the hospital. The drug prescription facility has been implemented later on, since January 2003, and its use is still increasing (currently by half of the hospital departments).

The hospital information system thus allows to install decision support systems that are activated whenever a prescription is issued and routinely to collect evaluation criteria of prescription practices. If possible and validated, the use of the hospital information system to evaluate care procedures will make it possible to collect data regularly, and routinely to assess methods for the improvement of care practices.

#### Organisation of quality assurance and system for recording undesirable events

With the aim of quality of care and preventing risks, the hospital is developing a system, based on the Intranet network, of declaration of undesirable events. This system must be able to record all undesirable events and incidents linked to the use of health products (drugs, medical equipment, blood products, etc.) and care (e.g. falls, lost files, bedsores, long waiting times) as well as those due to the patient environment (building, security, malevolence, etc.) and the job of health care professionals (accidental exposure to blood, chemicals, radiation, etc.).

When a health care professional decides to report an undesirable event, he or she must complete a dedicated form available on the Intranet with all relevant information. This incident form includes an item entitled "complications associated with anti-coagulants". When the doctor clicks on this item, a form specific to haemorrhagic accidents following anti-coagulant treatment appears (see form in appendix).

#### Prevention of thrombosis at GPEH

Among departments which already started to use the computerised drug order entry system, several (cardiology and vascular medicine departments) are heavy "consumers" of anti-thrombosis drugs (see Table 1).

In 2001, a working group representative of all the hospital departments described a group of procedures. This was done by critically reading articles published in the literature. The procedures concerned the preventive and curative indications of anticoagulants (OAT and heparins) in arterial thrombosis and venous thrombosis, and the way in which patients receiving anticoagulants are monitored and handled in cases of overdose. The procedure concerning curative OAT included a nomogram for adjusting doses of *fluindione *and *warfarin *based on previous doses and the INR (see Tables 2 and 3). These nomograms are currently available on the hospital's intranet, although they are not widely used. Their inclusion in a computerised prescription aid could increase their impact.

### Study aims

#### Main aims

1. To evaluate the effect on the frequency of overanticoagulation of the implementation in the computerised physician order entry system of two types of tool to adjust OAT doses (one personalised and one non-personalised).

2. To assess any advantages of using the personalised tool rather than the non-personalised tool.

#### Secondary aims

1. To evaluate the frequency of haemorrhagic accidents in the context of the study.

2. To evaluate the feasibility of long-term implementation of the intervention.

## Methods

### Experimental design

The study is an alternate time series experiment which consists of three successive six-month periods (timed to change with the changing of medical residents even though they will not be the only prescribers). Each phase will consist of:

• a two-month period without active support during which evaluation criteria will be collected (period A),

• a two-month period with non-personalised active support (period B),

• a two-month period with personalised active support (period C).

To limit the impact of a learning effect on appropriate OAT management practice within the department over time (possible for medical residents), the order of these three periods was determined by using a Latin square plan (see Figure). This experimental design can be considered valid for an impact study in this context whereas a randomised controlled design is difficult to apply with just one hospital [20].

### Participants

#### Patients

This study will include all the patients who are prescribed OAT for any indication and are hospitalised in clinical care departments where physicians are using the hospital information system to prescribe drugs.

The following table shows the number of INR examinations prescribed by these departments in a three-month period. It provides an estimation of the approximate proportion of overdoses among the INR that exceeded 2, which is supposed to be found in patients treated with OAT in these units.

#### Physicians

All doctors authorised to prescribe drugs in the participating departments will be included in the study: residents and fellows, registered and non registered university hospital doctors. Each six-month period will coincide with an internship semester.

### Intervention

To prescribe a drug using Dx-Care^®^, the doctor selects the required drug from an exhaustive list. This opens up a dialogue box in which the doctor types the dose, the frequency of intake and the mode of administration. From this window, it is possible to add a text comment or to consult particular protocols that have been defined by the departments.

It is planned to integrate two types of decision support systems into the computerised prescription program:

1) non-personalised active system: when the drug is selected a window automatically opens giving the prescribing the nomogram for the adjustment of OAT doses in the form of a table (see Tables 2 and 3).

2) personalised active system: when the drug is selected a window automatically opens suggesting a dose recommended according to the nomogram (taking into account the doses previously received by the patient and the patient's INR), together with a date for next INR control and an explication.

### Definition of endpoints

#### Overanticoagulation

Proportion of patient observation time with INR results > 4.5, assuming linear change of INRs.

#### Major haemorrhagic accidents

Intra-cranial haemorrhage or spontaneous haemorrhage necessitating surgery or a transfusion or decreasing haemoglobin concentration by more than 2 g/dl.

### Assessment of evaluation criteria

#### OAT overdose

The Netlab^® ^application allows biological laboratories to receive prescriptions and to return results. All of the INR results can be extracted from the Netlab^® ^database accompanied by information making it possible to identify the patient, the treatment and dose received, the prescribing doctor, the hospitalisation unit, the date the test was prescribed. Data about overdoses can therefore be collected systematically by regular database searches.

Furthermore, the storage of the information in a computerised tool will make it possible to determine previous doses and INR results each time a drug is prescribed.

#### Haemorrhagic accidents

When a health care professional decides to declare an undesirable event, he or she fills in a specific, pre-formatted form available on the Intranet. This form includes a list of events that must be declared at the GPEH.

The declaration form includes an item entitled "complication of haemorrhagic accidents". When the doctor clicks on this item, a specific form for the declaration of a haemorrhagic accident associated with anti-coagulant treatment appears (see form in appendix).

### Determination of sample size

The determination of the number of participants necessary requires the definition of the statistical unit of interest, information about the incidence of the evaluation criteria in the study population and a hypothesis about the efficiency of the intervention.

#### Statistical unit

In this study, the main aims are to guide each prescription and to reduce the number of anti-coagulant overdoses: the simplest statistical unit to study is therefore the INR result. This unit will be used to calculate the sample size.

This choice is not, however, perfect and the efficacy results will be presented using other indicators of the quality control of anti-coagulant treatments:

Given the low incidence of major haemorrhagic accidents (not currently measured at the GPEH but probably below 1%), it is not possible in this study to estimate the number of subjects necessary to demonstrate an effect of intervention on the "haemorrhagic accident" endpoint. Recording haemorrhagic accidents will give the frequency of such accidents, which will then be used for realistic estimates of power and sample size if further studies are carried out.

In previous studies evaluating the efficacy of tools to aid the prescription of OAT, the unit considered was not always the same, taking into account the number of INR per patient and the time between INR measurements to greater or lesser extents. The most recent studies considered the number of patient-days according to the method described by Rosendaal [15,16,21]. This method can also be used to calculate the rate of haemorrhagic events as a function of the number of patient-days for a given range of INR values.

We may also carry out an analysis for each prescribing doctor given that the intervention targets doctors directly. This will involve adjusting the effect of the intervention to the fact that intra-physician variability is *a priori *lower than inter-physician variability.

#### Number of INR measurements and predicted frequency of overdoses

During a six-month period (January to June 2004), 4 920 INRs were requested by the six departments which already routinely use the computerized physician order entry system. The frequency of overtreatment can approximately be estimated from the percentage of INR > 4.5 among INR >2. Among the 2620 INR > 2, 330 (12%) were higher than 4.5 (see Table 4). This frequency has been stable during these six months but differed considerably between departments (10% to 23%).

#### Hypothesis about the efficacy of the intervention

The number of INR tests necessary for a six-month period, with an α risk of 5% and a power of 80%, for the comparison of two percentages by classical methods (untreated group half the size of the treated group), for a basal incidence of the judgement criterion of 12% are and for the following hypotheses on relative reduction of the risk (RRR) of overdose, are:

• RRR 30%: 2500

• RRR 40%: 1300

• RRR 50%: 800

• RRR 60%: 500

Carrying out approximately 5000 tests over six months will make it possible to detect an intervention effect of less than 30% in this period. The experimental design includes three six-month periods and should thus ensure adequate power.

### Statistical analyses

Statistical analyses will be performed with the STATA statistical software (release 8, STATA corp, College station, Tex, USA)

Standard statistical tests will be used to compare the baseline characteristics of the departments and patients.

The main analysis concerns the effect of the intervention on the number of dangerously high INRs. The analysis will be carried out using a mixed effect analysis of variance model, in which the effect linked to the period will be considered fixed and that linked to the prescription tool will be considered random [22].

Rosendaal's method will be used to analyse the number of patient-days with INR over the target [21].

## Regulatory aspects

According to French policy, this study was exempt from medical ethics committee approval. The anti-coagulants being evaluated are prescribed as recommended by clinical studies validated within the GPEH. These recommendations are available on the hospital's Intranet and are thus accessible to all doctors. They conform to standard practices. Neither the patients nor the doctors will be randomised. The interventions are simply different means of giving valid information to physicians. Using funding from the PHRC (Hospital Clinical Research Program), we carried out two research studies related to this project. In the first (PHRC 95), an intervention aimed at modifying the way in which emergency department doctors handle ankle injuries, the study design was a randomised controlled study and the randomisation unit was the hospital [11]. In the second (PHRC 98), a computer-based decision-support system for the prevention of venous thromboses in orthopaedics, the experimental design was identical to that of our present study [12]. In both cases, the Ile de France branch of the Clinical Research Delegation considered that the project was exempt from medical ethics committee approval.

## List of abbreviations

OAT: Oral Anticoagulant Therapy

INR: International Normalized Ratio

GPEH: Georges Pompidou European Hospital

PHRC: Hospital Clinical Research Program

## Authors' contributions

IC and PD, conceived, wrote the protocol and prepared the manuscript.

GC is the statistical expert and performed the power calculations

GC and ABR revised the protocol and the manuscript.

## Pre-publication history

The pre-publication history for this paper can be accessed here:



## Figures and Tables

**Figure 1:**
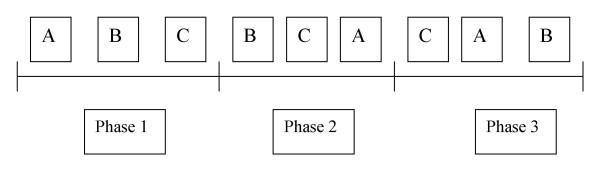
**Study Design. **A: Period without any decision support system; B: Period with non-personalised decision support system; C: Period with personalised decision support system

**Table 1:** Number of orders of *fluindione *during first semester 2004 in departments using computerized physician order entry system

**Department**	**January**	**February**	**March**	**April**	**May**	**June**	**Total**
Internal Medicine*		45	50	58	53	21	227
Immunology	17	11	2			1	31
Nephrology	16	32	35	32	2	34	151
Vascular Medicine – Hypertension	71	23	46	23	46	16	225
Cardiovascular Surgery	2	129	143	140	90	64	568
Cardiology (1 unit)*		6	55	38	28	66	193
Total	106	246	331	291	219	202	1395

*Department which started to use the computerised physicians' drug order entry system in February 2004. Only one unit of the three in the Cardiology department started to use it.

**Table 2:** Nomogram for adjustment of *fluindione *from [23]

**Day**	**INR**	**DOSE**
**D0**	< 1,2	20 mg
**D2**	1–1,4	30 mg
	1,5–1,7	25 mg
	1,8–2,3	20 mg
	2,4–3	15 mg
	>3	10 mg
**D4**	<1,8	+10 mg
	1,8–2,0	+4 mg
	2,1–2,5	unchanged
	2,6–3	dose > 20 mg: -5 mg
		dose ≤ 20 mg: inchangée
	>3	dose > 15 mg: -10 mg
		dose ≤ 15 mg: -5 mg
**D6**	<2,3	+5 mg
	2,3–3,5	unchanged
	>3,5	-5 mg

**Table 3:** Nomogram for adjustment of *warfarin *from [24]

**Day**	**INR**	**dose (mg)**			
		
		≤50 years	51–65 years	66–80 years	>80 years
**D0**	<1,4	10	9	7,5	6
**D1**	≤1,5	10	9	7,5	6
	≥1,6	0,5	0,5	0,5	0,5
**D2**	≤1,7	10	9	7,5	6
	1,8–2,3	5	4,5	4	3
	2,4–2,7	4	3,5	3	2
	2,8–3,1	3	2,5	2	1
	3,2–3,3	2	2	1,5	1
	3,4	1,5	1,5	1	1
	3,5	1	1	1	0,5
	3,6–4,0	0,5	0,5	0,5	0,5
	>4	0	0	0	0
**D3**	≤1,5	10–15	9–14	7,5–11	6–9
	1,6	8	7	6	5
	1,7–1,8	7	6	5	4
	1,9	6	5	4,5	3,5
	2,0–2,6	5	4,5	4	3
	2,7–3,0	4	3,5	3	2,5
	3,1–3,5	3,5	3	2,5	2
	3,6–4,0	3	2,5	2	1,5
	4,1–4,5	No pill, then 1–2	No pill, then 0,5–1,5	No pill, then 0,5–1,5	No pill, then 0,5–1,0

**Table 4:** Results of INR ordered during first semester 2004 in six departments

	Number of valid results	Number of INR > 2 (%)	No. of INR > 4.5 (% of INR > 2)
Internal Medicine*	525	294	45 (15.3%)
Immunology	28	17	4 (23.5%)
Nephrology	182	109	18 (16.5%)
Vascular Medicine – Hypertension	560	273	28 (10.3%)
Cardiovascular Surgery	1224	633	98 (15.5%)
Cardiology (1 unit)*	2401	1294	137 (10.3%)
Total	4920	2620	330 (12.6%)
